# C-C chemokine receptor type 5 links COVID-19, rheumatoid arthritis, and Hydroxychloroquine: in silico analysis

**DOI:** 10.1186/s41231-020-00066-x

**Published:** 2020-09-09

**Authors:** Mahmood Y. Hachim, Ibrahim Y. Hachim, Kashif Bin Naeem, Haifa Hannawi, Issa Al Salmi, Suad Hannawi

**Affiliations:** 1 College of Medicine, Mohammed bin Rashid University of Medicine and Health Sciences, Dubai, United Arab Emirates; 2 Clinical Sciences Department, College of Medicine, University of Sharjah, Sharjah, UAE; 3 Ministry of Health and Prevention (MOHAP), Dubai, UAE; 4 The Royal Hospital, Muscat, Oman

## Abstract

Patients with rheumatoid arthritis (RA) represent one of the fragile patient groups that might be susceptible to the critical form of the coronavirus disease − 19 (COVID-19). On the other side, RA patients have been found not to have an increased risk of COVID-19 infection. Moreover, some of the Disease-Modifying Anti-Rheumatic Drugs (DMARDS) commonly used to treat rheumatic diseases like Hydroxychloroquine (HCQ) were proposed as a potential therapy for COVID-19 with a lack of full understanding of their molecular mechanisms. This highlights the need for the discovery of common pathways that may link both diseases at the molecular side. In this research, we used the in silico approach to investigate the transcriptomic profile of RA synovium to identify shared molecular pathways with that of severe acute respiratory syndrome-corona virus-2 (SARS-COV-2) infected lung tissue. Our results showed upregulation of chemotactic factors, including CCL4, CCL8, and CCL11, that all shared CCR5 as their receptor, as a common derangement observed in both diseases; RA and COVID-19. Moreover, our results also highlighted a possible mechanism through which HCQ, which can be used as a monotherapy in mild RA or as one of the triple-DMARDs therapy (tDMARDs; methotrexate, sulphasalazine, and HCQ), might interfere with the COVID-19 infection. This might be achieved through the ability of HCQ to upregulate specific immune cell populations like activated natural killer (NK) cells, which were found to be significantly reduced in COVID-19 infection. In addition to its ability to block CCR5 rich immune cell recruitment that also was upregulated in the SARS-COV-2 infected lungs. This might explain some of the reports that showed beneficial effects.

## Introduction

Since the outbreak of Coronavirus disease-19 (COVID-19) disease, the clinical features of this disease showed significant variability between different subpopulations. Severe acute respiratory syndrome coronavirus 2, shortened to SARS-CoV-2, is the virus that causes COVID-19 disease [1]. Initially, patients with chronic conditions, as well as immunodeficiencies, were considered as high-risk groups patients for the development of the more severe form of the COVID-19 [2, 3]. Patients with rheumatoid arthritis (RA), a prevalent immune-mediated disease, are at higher risk of bacterial and viral infections due to its pathogenesis and the use of immunosuppressive agents as an RA treatment. As a result, RA patients represent one of those fragile patients groups that might be susceptible to the critical form of the COVID-19 disease [4–6].

Unexpectedly, recent reports showed that patients with RA have no increased risk of COVID-19 infection. Moreover, some of the Disease-Modifying Anti-Rheumatic (DMARDs) that commonly used to treat rheumatic diseases like Hydroxychloroquine (HCQ) were proposed as potential therapies for COVID-19 [7–10]. HCQ is used as monotherapy in mild RA cases, or it can be used as a combined treatment, particularly with methotrexate and sulphasalazine as Triple Disease Anti-Rheumatic Drugs (tDMARDs) regimen [11]. Several mechanisms were proposed for HCQ to produce its action, and this includes the anti-inflammatory effect through lysosomal acidification interference and phospholipase A2 inhibition [12, 13]. Also, HCQ was proposed to modulate the inflammatory response through its inhibition of the toll-like receptors signal as well as the T and B cell receptors leading to inhibition of their cytokine production, including the interleukin (IL)-1 and IL-6 [13, 14]. This cytokine inhibition was proposed as an essential mechanism that might explain the role of HCQ in reducing the cytokine storm critical in COVID-19 pathogenesis [15]. HCQ was also reported to inhibit viral replication [16].

The controversial results that recently linked to the efficacy of HCQ in COVID-19, in addition to the lack of full understanding of its molecular mechanisms, highlight the need for the discovery of common pathways that may link both diseases; COVID-19 and RA at the molecular side. This step is essential for the identification of possible targets that can block pathogenesis of RA and prevent severe forms of COVID-19. Also, it might help in identifying the predictive biomarkers that can help in more efficient patient stratification to predict COVID-19 patient’s responses to HCQ.

In this study, we used in silico approach to investigate the transcriptomic profile of RA synovium to identify shared molecular pathways with that of SARS-COV-2 infected lung tissue.

## Materials and methods

### RA synovium specific DEG

The Gene Expression Omnibus (GEO) public repository was used to retrieve the gene expression profile of synovial tissue from 33 RA, 26 osteoarthritis (OA) patients, and 20 healthy controls from three datasets (GSE55235, GSE55457, GSE55584) as previously reported [17]. Raw cell files were reanalyzed using AltAnalyze tool (20) and in house pipeline for normalization and filtration as previously described [18] to identify novel synovium related biomarkers.

### tDMARDs response in RA synovium

We used the publicly available synovial tissue transcriptomic data to compare the infiltration of the immune cells at baseline and after six months of tDMARDs to identify subgroups that might not respond well to tDMARDs. RNAseq dataset (GSE97165) of synovial biopsies taken from 19 early RA (defined as within 12 months of the onset of symptoms) patients at baseline and after six months of tDMARDs treatment were retrieved and reanalyzed.

### SARS-COV-2 and RA

RNAseq dataset (GSE147507) were retrieved using the GEO and used to identify Differentially Expressed Genes (DEGs) between infected and uninfected lung samples using BioJupies tools [19].

### Pathways and gene set enrichment

Differentially expressed genes between the subgroups were defined, and gene set enrichment analysis was performed to identify the underlying pathways in each group using BioJupies tools. The DEGs were explored for common pathways using Metascape online tool (http://metascape.org) [10].

### Estimating immune and stromal cells in the synovium

In order to achieve this goal, we used a recently available tool called ESTIMATE (Estimation of STromal and Immune cells in MAlignant Tumor tissues using Expression data) to estimate the difference in the infiltration of immune cells in healthy, OA and RA synovium. ESTIMATE R package was used to estimate the difference in immune cells’ infiltration between the three groups using their transcriptomic profile.

### Estimating infiltrating immune cells and their activation status in the synovium

The raw RNAseq data were used for in silico prediction of the immune cells’ infiltration of the synovial tissue using CIBERSORT analytical tool to evaluate the pre versus post tDMARDs changes in the immune population and/or activation status. Then, patients were divided according to the level of alteration in immune cells percentage after the treatment. The immune cells that express a higher level of the identified receptor were explored using the Database of Immune Cell Expression (DICE) project tool (https://dice-database.org/). The expression of the chemokine receptor was searched in a microarray dataset (GSE77298) of synovial biopsies of RA and healthy controls.

## Results

### RA synovium express genes related to immune cells activation, migration, signaling, and response to viruses

**Fig. 1:**
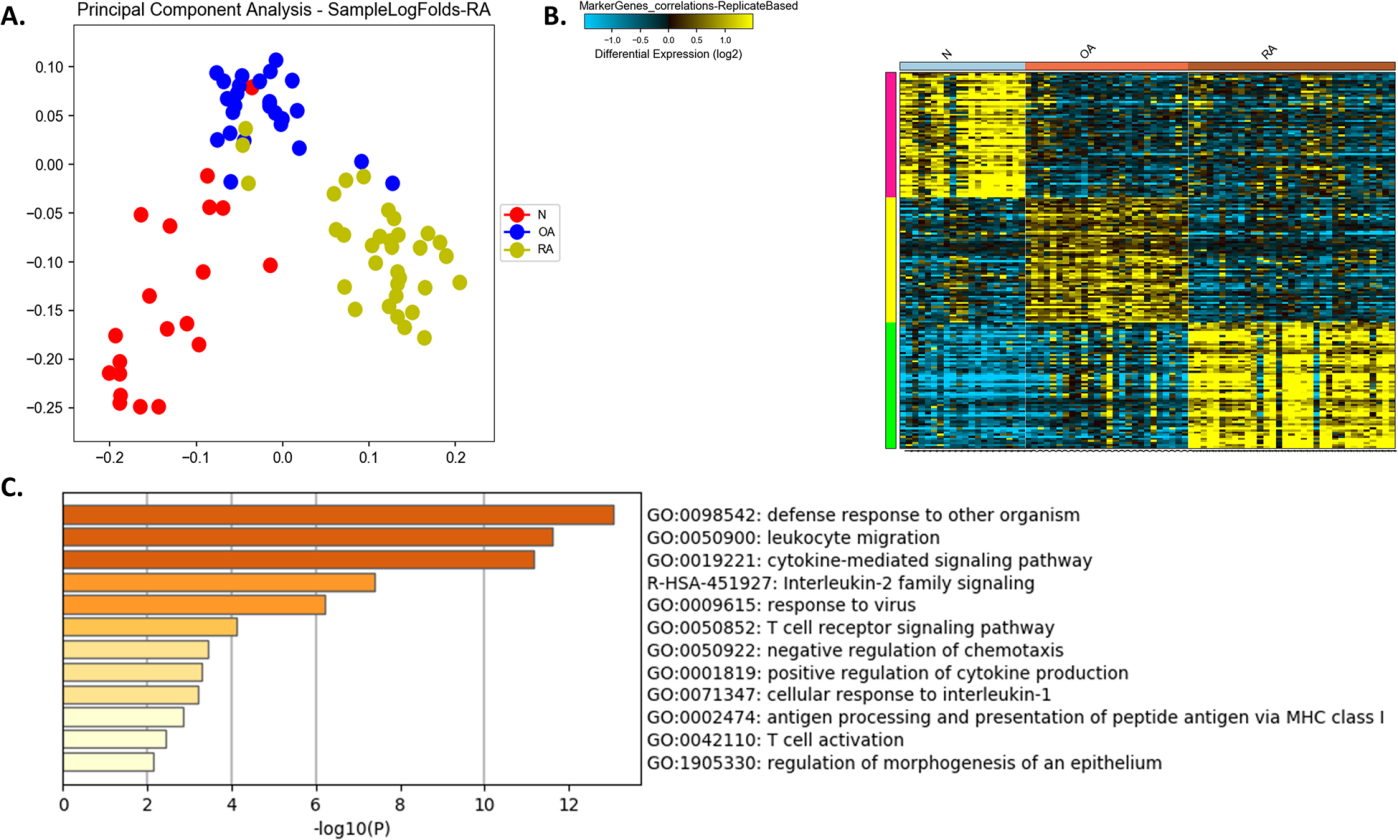
Comparison between the synovium transcriptomics profile of rheumatoid arthritis (RA) patients versus healthy controls (N) and osteoarthritis (OA). **a** principle component analysis (PCA) showing that the top selected DEGs can cluster the groups precisely **b** Heatmap of the top markers that can differentiate the three groups and **c** shows top pathways enriched in RA specific markers identified

For a better understanding of the RA disease pathogenesis, we reanalyze the gene expression profile of synovial tissue from 33 RA and compare to samples from 26 OA and 20 healthy controls. Our results showed that RA synovium expresses a specific signature that can differentiate it clearly from OA as well as healthy controls. This includes cytokine-mediated signaling pathway, positive regulation of cytokine production, Interleukin-2 family signaling, T cell receptor signaling pathway, leukocyte migration, negative regulation of chemotaxis, cellular response to interleukin-1, T cell activation, and regulation of morphogenesis of an epithelium. Moreover, pathways related to defense response to other organisms, antigen processing and presentation of peptide antigen via major histocompatibility complex (MHC) class I and response to the virus were also enriched specifically in RA synovium. (Fig. 1, Tables 1 and 2).

**Table 1:** Top Genes that are specific to healthy, OA, and RA synovium

ID	Markers Specific to Healthy		Markers Specific to OA		Markers Specific to RA	
	Probeset_id	Gene Name	Probeset_id	Gene Name	Probeset_id	Gene Name
1	204180_s_at	ZBTB43	204284_at	PPP1R3C	210538_s_at	BIRC3
2	204131_s_at	FOXO3	217963_s_at	NGFRAP1	217933_s_at	LAP3
3	213649_at	SFRS7	219197_s_at	SCUBE2	204279_at	PSMB9
4	222303_at		212256_at	GALNT10	216920_s_at	TARP
5	204243_at	RLF	203478_at	NDUFC1	211798_x_at	IGLJ3
6	222164_at	FGFR1	205330_at	MN1	217281_x_at	IGHV3–7
7	206359_at	SOCS3	218126_at	FAM82A2	209924_at	CCL18
8	201160_s_at	CSDA	210534_s_at	B9D1	217179_x_at	
9	209682_at	CBLB	202016_at	MEST	214973_x_at	IGHD
10	215330_at		204776_at	THBS4	209267_s_at	SLC39A8
11	219228_at	ZNF331	204797_s_at	EML1	218223_s_at	PLEKHO1
12	204748_at	PTGS2	201842_s_at	EFEMP1	211644_x_at	IGKV3–20
13	210764_s_at	CYR61	205364_at	ACOX2	205159_at	CSF2RB
14	218859_s_at	ESF1	214620_x_at	PAM	212956_at	TBC1D9
15	201465_s_at	JUN	210997_at	HGF	206247_at	MICB
16	220046_s_at	CCNL1	219953_s_at	C11orf17	217378_x_at	LOC100130100
17	200921_s_at	BTG1	208792_s_at	CLU	205488_at	GZMA
18	202768_at	FOSB	210302_s_at	MAB21L2	211643_x_at	
19	209184_s_at	IRS2	219182_at	FLJ22167	205569_at	LAMP3
20	213462_at	NPAS2	215913_s_at	GULP1	211637_x_at	IGHV4–4
21	200702_s_at	DDX24	37408_at	MRC2	205831_at	CD2
22	218880_at	FOSL2	213167_s_at	SLC5A3	213716_s_at	SECTM1
23	210094_s_at	PARD3	207326_at	BTC	209670_at	TRAC
24	207316_at	HAS1	207447_s_at	MGAT4C	206991_s_at	CCR5
25	210180_s_at	SFRS10	222125_s_at	P4HTM	214916_x_at	
26	208707_at	EIF5	206439_at	EPYC	216401_x_at	LOC652493
27	220266_s_at	KLF4	205127_at	PTGS1	214768_x_at	FAM20B
28	212501_at	CEBPB	218837_s_at	UBE2D4	217480_x_at	LOC339562
29	202340_x_at	NR4A1	209466_x_at	PTN	204891_s_at	LCK
30	211458_s_at	GABARAPL1	205150_s_at	KIAA0644	211645_x_at	
31	201473_at	JUNB	205898_at	CX3CR1	212314_at	KIAA0746
32	212384_at	BAT1	212713_at	MFAP4	205267_at	POU2AF1
33	200800_s_at	HSPA1A	205817_at	SIX1	219648_at	MREG
34	202014_at	PPP1R15A	201279_s_at	DAB2	210915_x_at	TRBC1
35	204622_x_at	NR4A2	206070_s_at	EPHA3	216576_x_at	IGKC
36	210852_s_at	AASS	205857_at	SLC18A2	217258_x_at	IGL@
37	202861_at	PER1	205638_at	BAI3	213915_at	NKG7
38	222162_s_at	ADAMTS1	206373_at	ZIC1	204613_at	PLCG2
39	215248_at	GRB10	220595_at	PDZRN4	221658_s_at	IL21R
40	214805_at	EIF4A1	218675_at	SLC22A17	202307_s_at	TAP1
41	201810_s_at	SH3BP5	217511_at	KAZALD1	203528_at	SEMA4D
42	202948_at	IL1R1	206726_at	PGDS	203828_s_at	IL32
43	212732_at	MEG3	204933_s_at	TNFRSF11B	201690_s_at	TPD52
44	217911_s_at	BAG3	211958_at	IGFBP5	214777_at	IGKV4–1
45	200768_s_at	MAT2A	221447_s_at	GLT8D2	216207_x_at	IGKV1D-13
46	221031_s_at	APOLD1	205833_s_at	PART1	206082_at	HCP5
47	202672_s_at	ATF3	203440_at	CDH2	208885_at	LCP1
48	212227_x_at	EIF1	204749_at	NAP1L3	1405_i_at	CCL5
49	203752_s_at	JUND	221029_s_at	WNT5B	M97935_3_at	STAT1
50	202431_s_at	MYC	207497_s_at	MS4A2	204116_at	IL2RG
51	213006_at	CEBPD	210372_s_at	TPD52L1	209374_s_at	IGHM
52	201531_at	ZFP36	210006_at	ABHD14A	209606_at	CYTIP
53	203140_at	BCL6	220076_at	ANKH	204533_at	CXCL10
54	36711_at	MAFF	213195_at	LOC201229	202270_at	GBP1
55	208869_s_at	GABARAPL1	204773_at	IL11RA	219386_s_at	SLAMF8
56	209681_at	SLC19A2	219416_at	SCARA3	205890_s_at	GABBR1
57	212665_at	TIPARP	206089_at	NELL1	205242_at	CXCL13
58	202284_s_at	CDKN1A	219561_at	COPZ2	206134_at	ADAMDEC1
59	209305_s_at	GADD45B	206480_at	LTC4S	203915_at	CXCL9
60	203574_at	NFIL3	205475_at	SCRG1	206513_at	AIM2

**Table 2:** Top Pathways enriched in the DEGs specific to RA compared to healthy and OA

Category	Term	Description	LogP	Log(q-value)	InTerm_InList	Symbols
GO Biological Processes	GO:0098542	defense response to other organism	−13.0634	−8.744	16/596	BIRC3, GBP1, IGHD, IGHM, IGKC, CXCL10, MICB, CXCL9, STAT1, AIM2, CXCL13, IGHV3–7, TRBC1, IGKV3–20, SLAMF8, IGLL5, CCR5, CSF2RB, GZMA, PLCG2, CCL5, POU2AF1, IGKV4–1, LCK, PSMB9, CD2, LCP1, TPD52, IL21R, TAP1, LAMP3, IGKV1D-13, IL2RG
GO Biological Processes	GO:0050900	leukocyte migration	−11.5986	−7.757	14/504	CD2, CCR5, IGHM, IGKC, CXCL10, LCK, CXCL9, CCL5, CCL18, CXCL13, IGHV3–7, IGKV4–1, IGKV3–20, SLAMF8, CSF2RB, IL2RG, IL21R, STAT1, PLCG2, SLC39A8, SEMA4D, PLEKHO1, FAM20B, ADAMDEC1, GABBR1, AIM2, BIRC3, NCOR2, GBP1
GO Biological Processes	GO:0019221	cytokine-mediated signaling pathway	−11.1576	−7.441	16/796	BIRC3, CCR5, CSF2RB, GBP1, IL2RG, CXCL10, LCP1, CXCL9, PSMB9, CCL5, CCL18, STAT1, IL32, AIM2, CXCL13, IL21R, LCK
Reactome Gene Sets	R-HSA-451927	Interleukin-2 family signaling	−7.38881	−4.517	5/44	CSF2RB, IL2RG, LCK, STAT1, IL21R, CCR5, GZMA, TAP1
GO Biological Processes	GO:0009615	response to virus	−6.22631	−3.606	8/334	BIRC3, GBP1, CXCL10, MICB, CXCL9, CCL5, STAT1, AIM2, PSMB9, CCL18, PLCG2, SLAMF8, LCK, LAMP3, CCR5, TAP1
GO Biological Processes	GO:0050852	T cell receptor signaling pathway	−4.11131	− 1.834	5/202	GBP1, LCK, PLCG2, PSMB9, TRBC1, CD2, TPD52, SLAMF8, BIRC3, STAT1, MICB
GO Biological Processes	GO:0050922	negative regulation of chemotaxis	−3.43346	−1.270	3/64	SEMA4D, CXCL13, SLAMF8, GBP1, LCK, CCL5, CYTIP, ADAMDEC1, CCL18, AIM2, TBC1D9, SLC39A8, CXCL10, PLCG2, MICB
GO Biological Processes	GO:0001819	positive regulation of cytokine production	−3.28901	−1.157	6/467	BIRC3, CD2, IGHD, PLCG2, STAT1, AIM2, GBP1, CCL5, LCP1
GO Biological Processes	GO:0071347	cellular response to interleukin-1	−3.19668	−1.073	4/180	GBP1, PSMB9, CCL5, CCL18, STAT1
GO Biological Processes	GO:0002474	antigen processing and presentation of peptide antigen via MHC class I	−2.85603	−0.782	3/101	MICB, PSMB9, TAP1
GO Biological Processes	GO:0042110	T cell activation	−2.4476	−0.425	5/472	CD2, LCK, LCP1, MICB, CCL5
GO Biological Processes	GO:1905330	regulation of morphogenesis of an epithelium	−2.14316	−0.163	3/181	CXCL10, PSMB9, STAT1, LCK, NCOR2

### RA synovium express higher CCL5 and its receptor CCR5

Next and in order to investigate the role of the main cytokines that control the immune response including cell number, activation, maturation, differentiation, and migration, we filtered the top DEGs between the three groups (healthy, OA, and RA) to look for chemokines and interleukins only. Interestingly, RA synovium showed significantly higher expression of important chemokines ligands (CCL18, CXCL9, CXCL10, CXCL13 CCL5, and its receptor CCR5). Moreover, RA synovium expresses higher interleukins related genes (IL21R, IL32, IL2RG) (Table 3).

**Table 3:** Top chemokine and interleukins related genes in the DEGs specific to RA compared to healthy and OA

Groups	Symbol	log_fold-OA_vs_N	adjp-OA_vs_N	log_fold-RA_vs_N	adjp-RA_vs_N	log_fold-RA_vs_OA	adjp-RA_vs_OA	ANOVA-rawp	ANOVA-adjp	largest fold
Healthy	IL1R1	−1.04318	3.14E-06	−1.08606	4.87E-08	−0.04288	0.835425	1.84E-12	2.15E-10	1.086057
OA	CX3CR1	2.285902	9.5E-11	1.231936	2.49E-05	−1.05397	1.18E-05	3.35E-15	1.12E-12	2.285902
OA	IL11RA	0.938146	1.54E-05	−0.08216	0.711224	−1.02031	4.32E-10	1.52E-11	1.25E-09	1.020306
RA	CCL5	0.164174	0.521514	1.630079	3.64E-09	1.465904	1.42E-09	1.17E-15	4.92E-13	1.630079
RA	CCR5	0.34399	0.043302	1.251077	1.01E-10	0.907087	9.01E-08	2.75E-15	9.57E-13	1.251077
RA	CCL18	0.355375	0.432754	2.165572	3.85E-09	1.810197	6.47E-09	1.86E-13	3.26E-11	2.165572
RA	CXCL9	0.177374	0.57414	2.881994	7.72E-11	2.704619	7.48E-13	8.75E-21	6.58E-17	2.881994
RA	CXCL10	0.462931	0.022287	2.326323	1.16E-10	1.863391	5.94E-09	1.66E-17	1.91E-14	2.326323
RA	CXCL13	0.46977	0.094643	3.919964	4.83E-12	3.450194	5.31E-11	8.86E-21	6.58E-17	3.919964
RA	IL2RG	0.212245	0.311266	1.6011	7.46E-10	1.388855	8.68E-10	1.23E-16	8.85E-14	1.6011
RA	IL32	0.40024	0.056249	1.659554	3.32E-10	1.259314	4.85E-09	4.25E-16	2.37E-13	1.659554
RA	IL21R	0.08408	0.495735	1.135477	1.03E-07	1.051397	2.02E-08	9.69E-15	2.58E-12	1.135477

### RA synovium showed a higher infiltration of plasma cells, CD4 memory T cells, and gamma delta T cells but less dendritic and activated NK cells

**Fig. 2:**
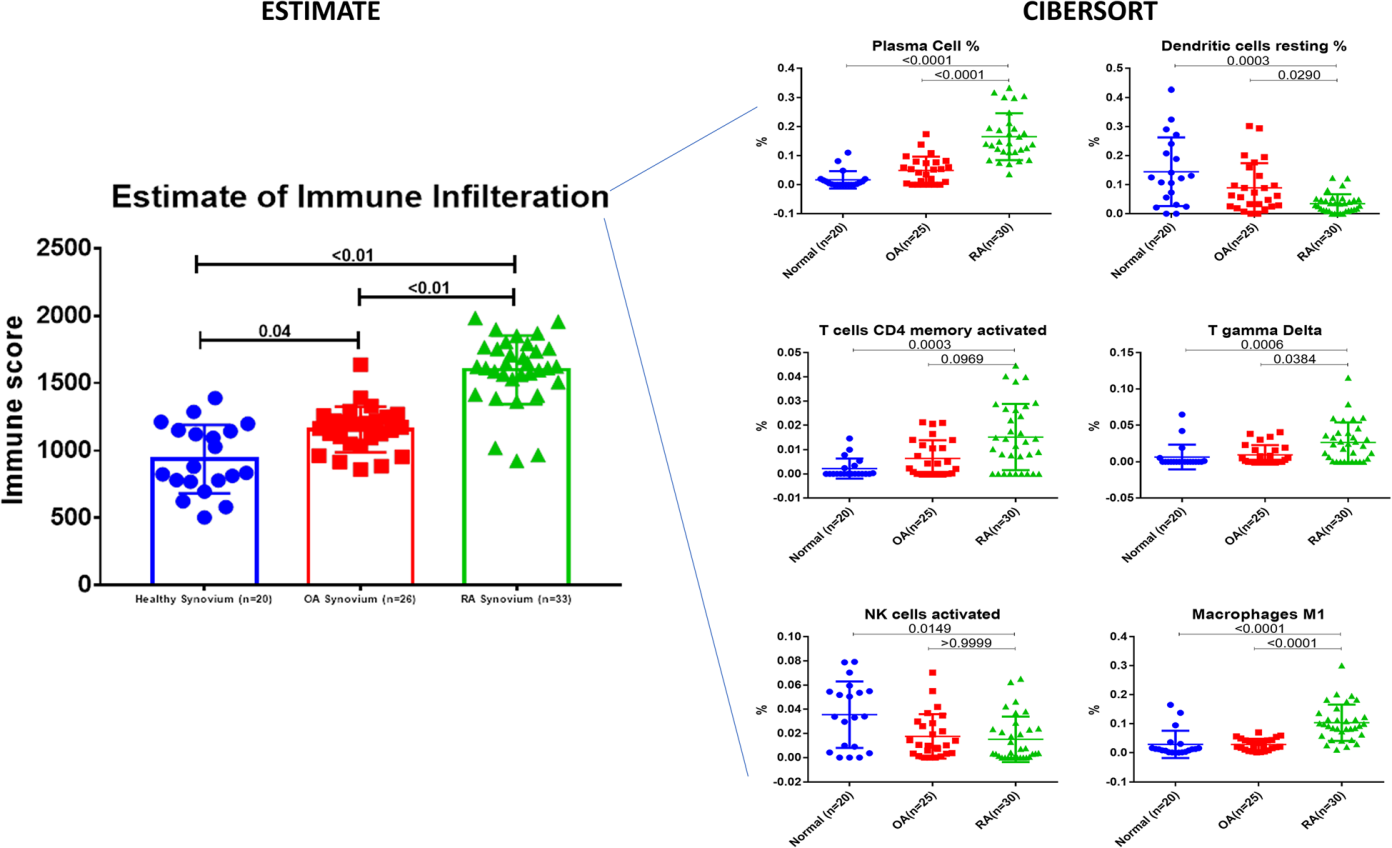
Estimating immune cells infiltration in the synovium using transcriptomics profile of rheumatoid arthritis (RA) patients versus healthy controls (N) and osteoarthritis (OA). We used the ESTIMATE tool to estimate the difference in the infiltration of immune cells in healthy, OA, and RA synovium using their transcriptomic profile. The raw RNAseq data were used for in silico prediction of the immune cells’ infiltration of the synovial tissue using CIBERSORT analytical tool to evaluate changes in the immune population and/or activation status between the groups

In order to decipher the effect of infiltrating immune cells to the synovium and their status of activation, which might mask the local gene expression and can explain the dynamics of immune cells in disease pathophysiology, we explored the immune infiltration using in silico tools. RA synovium showed a significantly higher level of infiltrating immune cells compared to OA and healthy controls confirming the DEGs and pathways enrichment results. Specifically, RA synovium showed higher infiltration of plasma cells, CD4 memory T cells, and gamma delta T cells but less dendritic and activated NK cells (Fig. 2).

### SARS-COV-2 infected lungs express more CCL4, CCL8, and CCL11 that share CCR5 as a common receptor

**Fig. 3:**
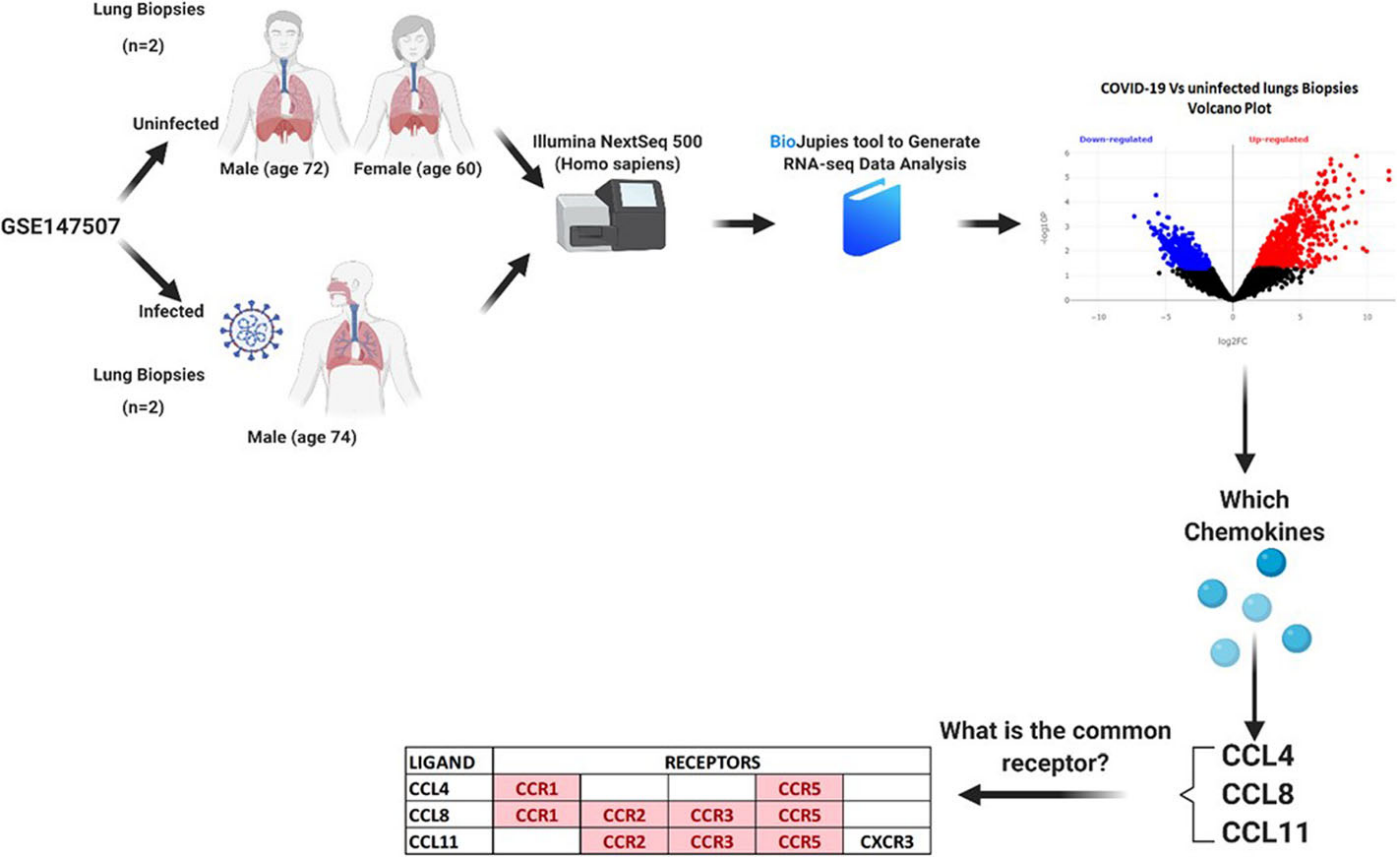
Flowchart for identification of DEGs between SARS-CoV-2 infected and uninfected lung samples using RNAseq dataset (GSE147507) retrieved from GEO using BioJupies tools. The flow of transcriptomics reanalysis, identification of chemokines, their common receptors, and immune cells with high receptor are summarized

Next, we tried to understand some of the molecular mechanisms involved in SARS-COV-2 pathogenesis with potential interaction with the mechanisms and pathways involved in RA. Eighty-four DEGs were identified between uninfected and COVID-19 infected lung samples. These DEGs were enriched in pathways specific to (response to the virus, response to interferon, leukocyte activation, and chemotaxis) Interestingly, SARS-COV-2 infected lungs express more CCL4, CCL8, and CCL11; the three ligands shared the same receptor, which is CCR5 (Fig. 3). Top immune cells that express CCR5 were CD4 T memory T reg cells, Th17, Th1, and monocytes.

### tDMARDs treatment in early RA increase synovial activated natural killers and resting mast cells but decrease plasma cells and M1 macrophages

**Fig. 4:**
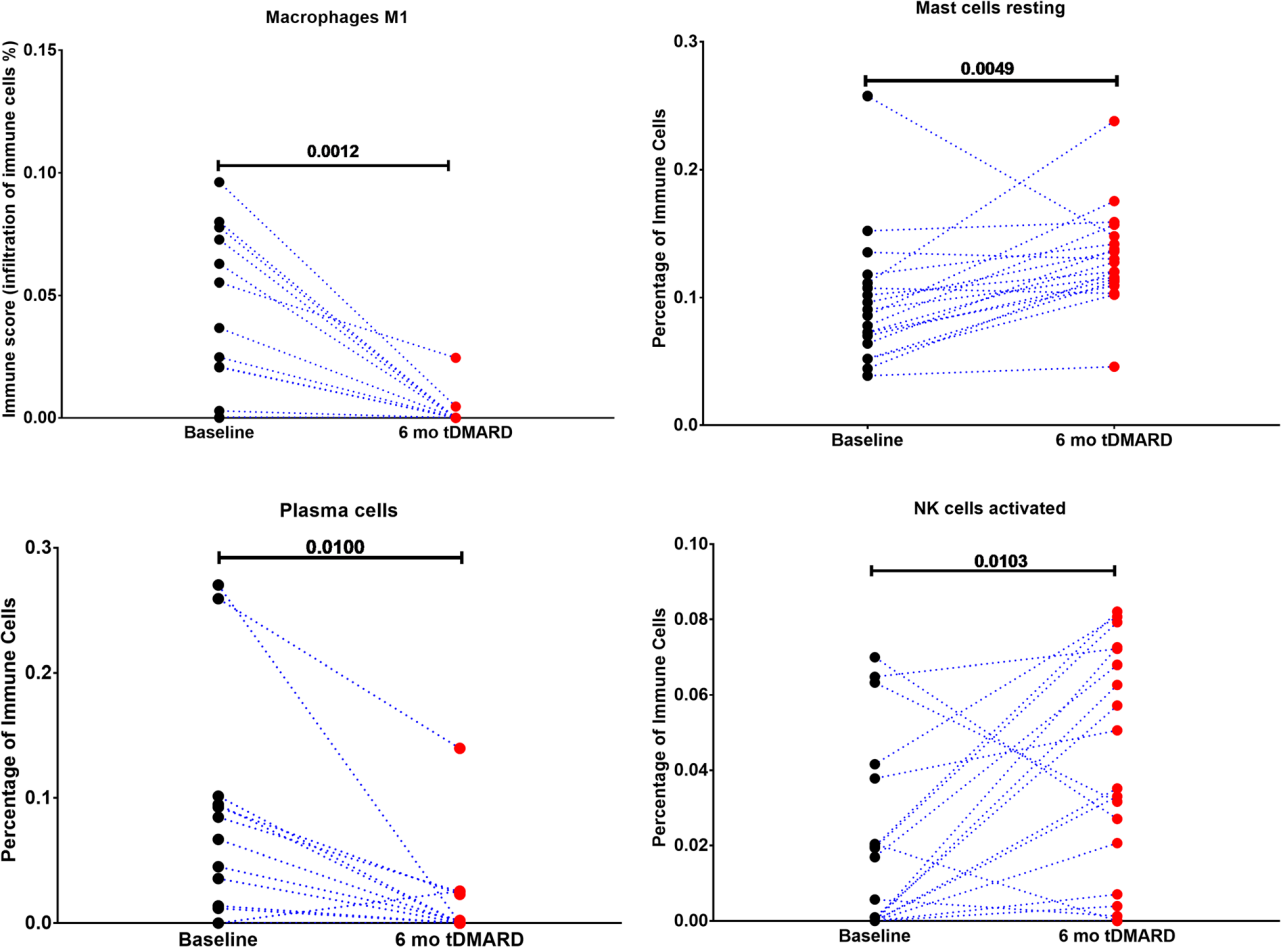
Effect of tDMARDs Treatment In Early RA synovial immune cells profile. We used the publicly available synovial tissue transcriptomic data to compare the infiltration of the immune cells at baseline and after six months of tDMARDs to identify subgroups that might not respond well to tDMARDs. RNAseq dataset (GSE97165) of synovial biopsies taken from 19 early RA (defined as within 12 months of the onset of symptoms) patients at baseline and after six months of tDMARDs treatment were retrieved and reanalyzed. ANOVA test was used

Next, we tried to investigate the effect of tDMARDs on immune modulation, which might improve our understanding of its role in the treatment of RA as well as other diseases like COVID-19 infection. To achieve this, we investigated the effect of the treatment of tDMARDs on different immune cell populations of the synovium. Our results showed that four immune cell populations were significantly changed after six months of tDMARDs. This includes the resting mast cells and activated NK cells that were shown to be increased by 84 and 74% of patients, respectively. On the other hand, M1 macrophages and plasma cells were decreased after treatment in 68 and 58% of patients, respectively (Fig. 4).

### DMARDs can block RA pathogenic CCR5 rich immune cell recruitment

**Fig. 5:**
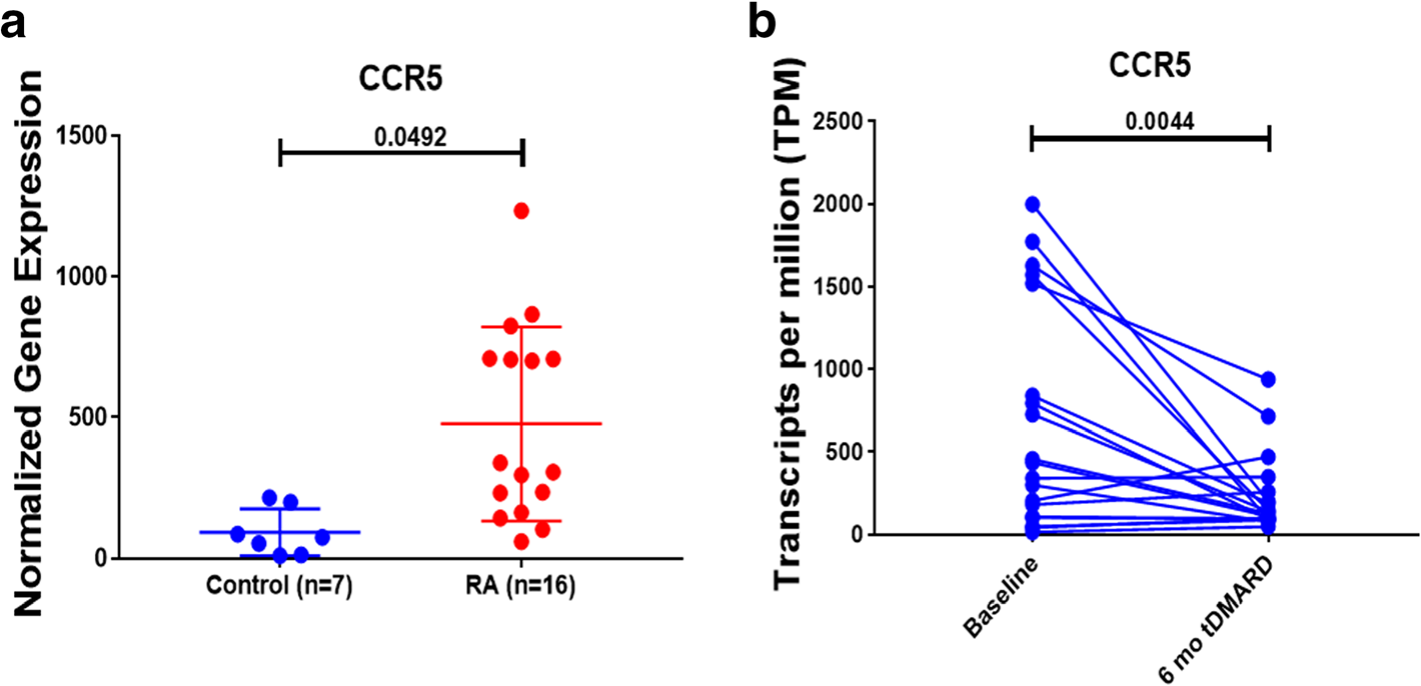
CCR5 expression in synovial biopsies of RA and control and CCR5 expression at baseline and after six months of tDMARDs treatment. The expression of the chemokine receptor was searched in a microarray dataset (GSE77298) of synovial biopsies of RA and healthy controls. A paired T-test was used for comparison

Further analysis confirmed our previous finding that CCR5 was significantly upregulated in RA compared to healthy controls synovium (*p* = 0.04), Fig (5a). Moreover, our results also showed that this receptor was dramatically downregulated after six months of tDMARDs treatment (*p* = 0.004), as shown in Fig. (5b). Those results highlighted a possible beneficiary effect of DMARDs in patients with COVID-19, through its ability to block CCR5 rich immune cell recruitment that we already found to be upregulated in the SARS-COV-2 infected lungs.

## Discussion

Since the outbreak of COVID-19 infection, it was evident that this disease had a variable clinical impact on different subpopulations [2, 3]. Due to the immune dysregulation as well as the use of immune-modulating treatments, patients with rheumatic diseases were considered among the fragile subpopulations that might suffer from the more aggressive form of COVID-19 [4–6]. Interestingly, a group of disease-modifying anti-rheumatic drugs (DMARDS), including HCQ and IL6 inhibitors such as tocilizumab, was also proposed as a possible therapeutic option to treat COVID-19 patients [20]. However, the mechanisms through which those agents produce their effect is not fully understood.

Chloroquine and hydroxychloroquine showed antiviral characteristics in vitro, and some reports showed their efficacy in the treatment of COVID-19 [8]. It is suggested that these drugs interfere with lysosomal activity, membrane stability, signaling pathways, and immune-related transcriptional activity [21].

Therefore, a better understanding of the relationship between RA and its associated therapies and COVID-19 disease might help to improve the response to COVID-19 pandemic. Our results here highlight a possible link between RA and COVID-19, which might explain the molecular basis of the benefits of some of the DMARDS used for treating COVID-19 infection.

Indeed, SARS-COV-2 infected lungs showed upregulation of chemotactic factors, including CCL4, CCL8, and CCL11, that all shared CCR5 as their receptor. This receptor is mainly expressed in the CD4 T memory, T reg cells, Th17, Th1, and monocytes.

Recent reports showed the importance of this receptor in the pathogenesis of RA. Indeed, CCR5 were found to be highly expressed in RA synovium, in addition to massive infiltration of the synovium with T helper cell type 1 inflammatory cell [22].

Our results showed that lungs infected with SARS-CoV-2 express higher levels of CCL4, CCL8, and CCL11. CCL4 exhibit chemoattractive ability towards different cell types, including immune cells, and coronary endothelial cells [23]. CCL4 and its receptor CCR5 were reported to be significantly induced in the infarct myocardium, vulnerable atherosclerosis plaques, advanced atherosclerotic lesions, and to be associated with a higher risk of stroke and cardiovascular events [23]. The other chemokines ligand CCL8 is known to recruits further neutrophils to the infarct to release MMPs and soluble IL-6 [24]. CCL11 bind CCR3 to stimulates the migration of immune cells like neutrophils [25] and was shown to recruit such cells to the heart and contribute to myocardial fibrosis [26].

The pathogenesis of RA is suggested to involve Th1-type T cells that preferentially express CCR5 where its chemokines ligands (macrophage inflammatory protein (Mip)-1α, CCL3; and Mip-1β, CCL4) participate in selective recruitment of CCR5 + CXCR3+ T cells to the inflamed synovium [27]. The infiltration of such IFN-γ secreting CCR5 + CD4+ T cells into the RA joint cavity is regulated by the synovial microenvironment [28]. On the other hand, CCR5 silencing suppresses inflammatory response in RA by inhibiting synovial cell viability but promoting apoptosis [29]. Another source of CCR5 in RA are Vδ2 T cells which infiltrated into the synovium under the influence of high levels of TNF-α [30]. Moreover, an in vivo model using a non-functional form of the CCR5 receptor (CCR5-Δ32) was shown to protect against RA [31, 32]. Carriers of the CCR5-Δ32 allele were at a significantly higher frequency in non-severe compared to severe patients making it a genetic marker related to the severity of RA [33].

In COVID-19 patients, disruption of the CCL5-CCR5 axis through CCR5 blocking antibody leronlimab was shown to reduce plasma IL-6, and SARS-CoV-2 plasma viremia [34]. For that reason, leronlimab is currently under investigation in a Phase2b/3 for severely ill COVID-19 patients [35]. Interestingly, the CCR5 Δ32 allele was found to be an important genetic marker of SARS-CoV-2 related death [36].

The similarity that we observe here in the pathogenesis of both diseases might provide evidence about the molecular pathways through which many of the commonly used drugs for RA treatment are proposed to have benefits in COVID-19 management [4].

Another observation we notice here is the finding that the tDMARDs used for RA treatment was able to significantly upregulate some immune cell populations, including resting mast cells and activated NK cells. The recent observation that during the COVID-19 infection, the main lymphocyte populations, including NK cells, were remarkably decreased, and this decrease was more prominent in the severe cases of COVID-19 infection compared to mild cases as well as healthy controls [37, 38]. Moreover, another report also revealed that NK cells, in addition to the CD8+, were found to be important in modulating the anti-COVID-19 response [39].

This might explain the recent findings that patients with chronic arthritis treated with different forms of DMARD showed no evidence of increased risk of life-threatening or respiratory complications following the COVID-19 infection compared to the general population [4].

On the other hand, our reanalysis showed that tDMARDs significantly decrease the M1 macrophages and plasma cells, as shown in Fig. 4. It is known that the number and the level of activation of inflamed synovial macrophages correlate significantly with the severity of RA [40]. In RA, synovium can forms a niche for potentially autoreactive—B cells and plasma cells that play a central role in RA pathogensis [41]. The ability of tDMARDs to block these cells can explain its anti-RA effects.

Lung macrophages in severe COVID-19 infection orchestrate local inflammation by recruiting inflammatory monocytic cells and neutrophils, whereas, in moderate COVID-19 infection, macrophages produce more T cell-attracting chemokines [42]. SARS-CoV-2 infection of alveolar macrophage can drive the “cytokine storm” that further damages multiple organs other than the lung, as in the case of heart and kidney [43].

During SARS-CoV-2 infections, immune cell subsets change, and among the B cells, the plasma cells increased remarkably, whereas the naïve B cells decreased [44]. Interestingly, one of the characteristics of the formation of SARS-CoV-2 anti-virus antibodies in a trial to limit viral replication is that these protective antibodies will cause friendly damage by the binding of the virus-Ab complex to FcR on monocytes/macrophages induces pro-inflammatory responses that end up with the accumulation of pro-inflammatory M1 macrophages in the lungs escalating lung injury [45].

The ability of tDMARDs to significantly decrease the M1 macrophages and plasma cells can suggest that such drugs can be beneficial only in those who develop severe to moderate disease and have secondary antiviral antibodies, and this can explain why not all patients receiving such therapy are benefited from them.

**Fig. 6:**
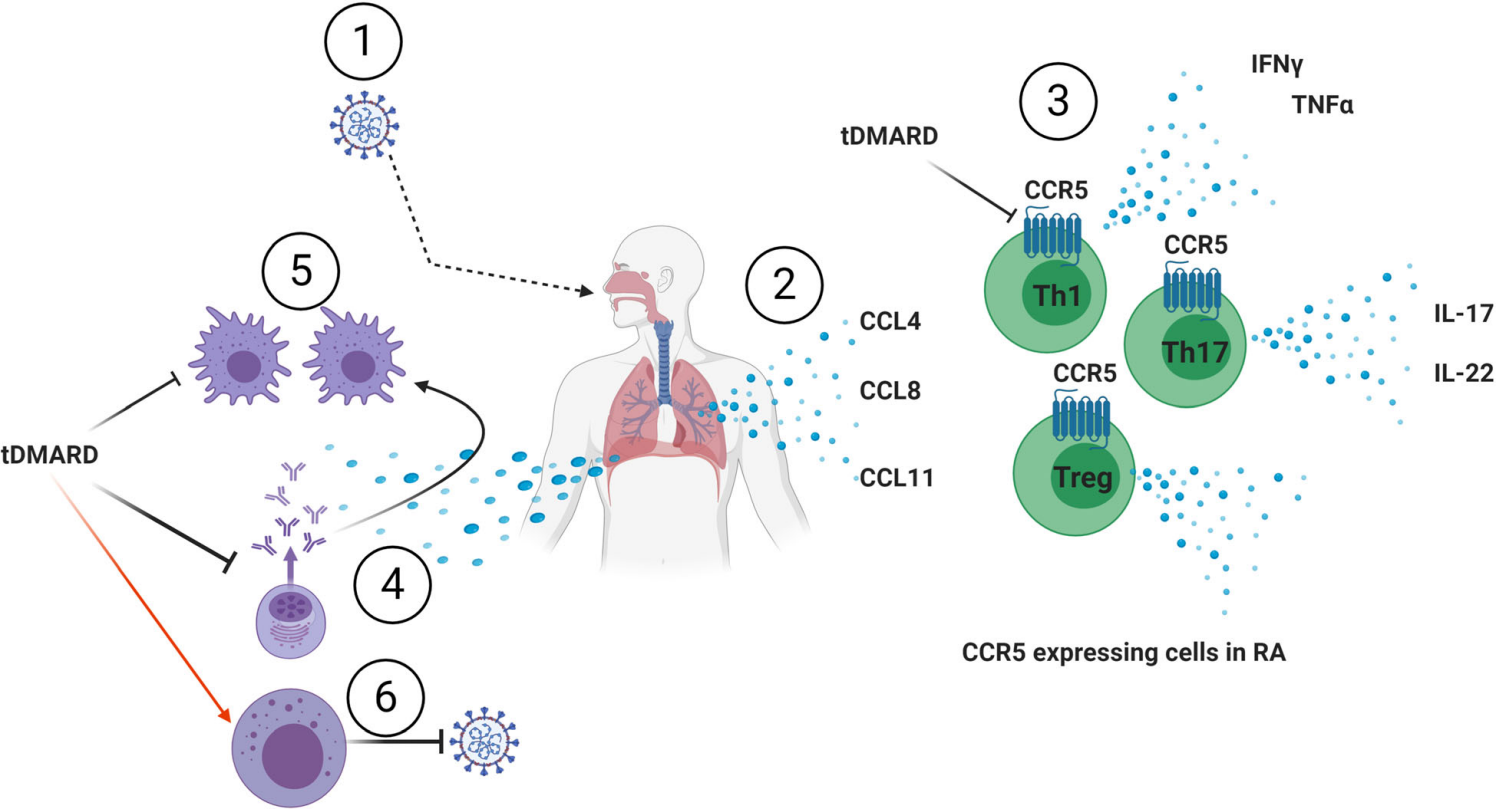
A working hypothesis for tDMARDs and COVID-19 interactions. The possible role of (1) SARS-COV-2 infected lungs (2) chemokines in recruiting (3) CCR5 rich immune cells. Epithelial cells secrete three chemokines that recruit immune cells that stimulate Th17 and Th1 profile to kill the virus but recruit inflammatory to the area. Infected epithelium can stimulate (4) plasma cells to secrete antiviral Ab that can (5) stimulate local macrophages to have an inflammatory M1 profile. tDMARDs can be helpful in the COVID-19 scenario by blocking CCR5 expression on immune cells plus inhibiting plasma amd M1 macrophages while enhancing NK cells to kill the virus

In contrast, our results demonstrate a possible mechanism through which HCQ as a member of DMARDs might help in the management of COVID-19 infection, Fig (6). The possible role SARS-COV-2 infected lungs chemokines in recruiting CCR5 rich immune cells. Epithelial cells secrete three chemokines that recruit immune cells that stimulate Th17 and Th1 profile to kill the virus but recruit inflammatory to the area. Infected epithelium can stimulate plasma cells to secrete antiviral Ab that stimulates local macrophages to have an inflammatory M1 profile. tDMARDs can be helpful in the COVID-19 scenario by blocking CCR5 expression on immune cells plus inhibiting plasma and M1 macrophages while enhancing NK cells to kill the virus.

Some issues to be considered carefully based on our results is that tDMARDs effect on CCR5 can inhibit Regulatory T (Treg) recruitment, which is required to inhibit the immune response and were reported to be reduced in severe COVID-19 patients [46]. Such an effect of HCQ might hamper innate and adaptive antiviral immune responses leading to growing uncertainty about these agents for the treatment of COVID-19 [47].

## Conclusion

In summary, our results highlight common pathways that are involved in the pathogenesis of RA as well as COVID-19. Those pathways might represent ideal targets for the discovery of more efficient and targeted therapeutic options to treat RA and COVID-19. Besides, it might help to improve our understanding of the mechanisms through which some of the medications are already used to treat COVID-19 infection, including the HCQ.

## Data Availability

Data generated in the study are included in the tables.

## Abbreviations

C-C: chemokine
COVID-19: coronavirus disease − 19
RA: rheumatoid arthritis
SARS-COV-2: severe acute respiratory syndrome-corona virus-2
tDMARDs: triple-DMARDs therapy; methotrexate, sulphasalazine, and HCQ
NK: natural killer cells
DMARDs: Disease-Modifying Anti-Rheumatic Drugs
HCQ: Hydroxychloroquine
IL: interleukin
GEO: Gene Expression Omnibus
OA: osteoarthritis
DEGs: Differentially Expressed Genes
ESTIMATE: Estimation of STromal and Immune cells in MAlignant Tumor tissues using Expression data
DICE: Database of Immune Cell Expression
MHC: major histocompatibility complex

## References

[1] Lai C-C, et al. Severe acute respiratory syndrome coronavirus 2 (SARS-CoV-2) and coronavirus disease-2019 (COVID-19): the epidemic and the challenges. Int J Antimicrob Agents. 2020;55(3):105924. 10.1016/j.ijantimicag.2020.105924 PMC7127800 32081636

[2] Guan WJ (2020). Clinical characteristics of coronavirus disease 2019 in China. N Engl J Med.

[3] Wu Z, McGoogan JM. Characteristics of and important lessons from the coronavirus disease 2019 (COVID-19) outbreak in China: summary of a report of 72314 cases from the Chinese Center for Disease Control and Prevention. JAMA. 2020. 10.1001/jama.2020.2648. [Published online ahead of print, 2020 Feb 24]. 10.1001/jama.2020.2648 32091533

[4] Favalli EG (2020). COVID-19 infection and rheumatoid arthritis: Faraway, so close!. Autoimmun Rev.

[5] Hsu CY (2019). Comparing the burdens of opportunistic infections among patients with systemic rheumatic diseases: a nationally representative cohort study. Arthritis Res Ther.

[6] Mehta B (2019). Serious infection risk in rheumatoid arthritis compared with non-inflammatory rheumatic and musculoskeletal diseases: a US national cohort study. RMD Open.

[7] Kim AHJ, Sparks JA, Liew JW, et al. A rush to judgment? Rapid reporting and dissemination of results and its consequences regarding the use of hydroxychloroquine for COVID-19. Ann Intern Med. 2020;172(12):819–21. 10.7326/M20-1223. 10.7326/M20-1223 PMC7138335 32227189

[8] Meo SA, Klonoff DC, Akram J (2020). Efficacy of chloroquine and hydroxychloroquine in the treatment of COVID-19. Eur Rev Med Pharmacol Sci.

[9] Million M, Lagier JC, Gautret P, et al. Early treatment of COVID-19 patients with hydroxychloroquine and azithromycin: a retrospective analysis of 1061 cases in Marseille, France. Travel Med Infect Dis. 2020;35:101738. 10.1016/j.tmaid.2020.101738. 10.1016/j.tmaid.2020.101738 PMC7199729 32387409

[10] Luo P (2020). Tocilizumab treatment in COVID-19: a single center experience. J Med Virol.

[11] Dale J (2007). Combination therapy for rheumatoid arthritis: methotrexate and sulfasalazine together or with other DMARDs. Nat Clin Pract Rheumatol.

[12] Loffler BM (1985). Effects of antimalarial drugs on phospholipase a and lysophospholipase activities in plasma membrane, mitochondrial, microsomal and cytosolic subcellular fractions of rat liver. Biochim Biophys Acta.

[13] Sperber K (1993). Selective regulation of cytokine secretion by hydroxychloroquine: inhibition of interleukin 1 alpha (IL-1-alpha) and IL-6 in human monocytes and T cells. J Rheumatol.

[14] Ben-Zvi I (2012). Hydroxychloroquine: from malaria to autoimmunity. Clin Rev Allergy Immunol.

[15] Zhao M (2020). Cytokine storm and immunomodulatory therapy in COVID-19: role of chloroquine and anti-IL-6 monoclonal antibodies. Int J Antimicrob Agents.

[16] Sinha N, Balayla G. Hydroxychloroquine and covid-19. Postgrad Med J. 2020. 10.1136/postgradmedj-2020-137785. 10.1136/postgradmedj-2020-137785 PMC10016880 32295814

[17] Hachim M (2019). Estimating the infiltration of immune cells in Synovium of rheumatoid arthritis compared to osteoarthritis and healthy control using Transcriptomic profiling.

[18] Hachim MY (2019). Identifying Asthma genetic signature patterns by mining Gene Expression BIG Datasets using Image Filtering Algorithms. 2019 IEEE International Conference on Imaging Systems and Techniques (IST).

[19] Torre D, Lachmann A, Ma’ayan A (2018). BioJupies: Automated Generation of Interactive Notebooks for RNA-Seq Data Analysis in the Cloud. Cell Systems.

[20] Zhong J, et al. The immunology of COVID-19: is immune modulation an option for treatment. Lancet Rheumatol. 2020;2(7):e428–36. 10.1016/S2665-9913(20)30120-X PMC7239618 32835246

[21] Schrezenmeier E, Dörner T (2020). Mechanisms of action of hydroxychloroquine and chloroquine: implications for rheumatology. Nat Rev Rheumatol.

[22] Mellado M (2015). T cell migration in rheumatoid arthritis. Front Immunol.

[23] Chang T-T, Chen J-W (2016). Emerging role of chemokine CC motif ligand 4 related mechanisms in diabetes mellitus and cardiovascular disease: friends or foes?. Cardiovasc Diabetol.

[24] Jones DP, True HD, Patel J (2017). Leukocyte trafficking in cardiovascular disease: insights from experimental models. Mediat Inflamm.

[25] Kindstedt E (2017). CCL11, a novel mediator of inflammatory bone resorption. Sci Rep.

[26] Zweifel M (2010). Eotaxin/CCL11 levels correlate with myocardial fibrosis and mast cell density in native and transplanted rat hearts. Transplant Proc.

[27] Patel DD, Zachariah JP, Whichard LP (2001). CXCR3 and CCR5 ligands in rheumatoid arthritis Synovium. Clin Immunol.

[28] WANG CR, LIU MF (2003). Regulation of CCR5 expression and MIP-1α production in CD4+ T cells from patients with rheumatoid arthritis. Clin Exp Immunol.

[29] Lan Y-Y, Wang Y-Q, Liu Y (2019). CCR5 silencing reduces inflammatory response, inhibits viability, and promotes apoptosis of synovial cells in rat models of rheumatoid arthritis through the MAPK signaling pathway. J Cell Physiol.

[30] Mo W-X (2017). Chemotaxis of Vδ2 T cells to the joints contributes to the pathogenesis of rheumatoid arthritis. Ann Rheum Dis.

[31] Zhou Y (1998). Impaired macrophage function and enhanced T cell-dependent immune response in mice lacking CCR5, the mouse homologue of the major HIV-1 coreceptor. J Immunol.

[32] Takeuchi T, Kameda H (2012). What is the future of CCR5 antagonists in rheumatoid arthritis?. Arthritis Res Ther.

[33] Zapico I (2000). CCR5 (chemokine receptor-5) DNA-polymorphism influences the severity of rheumatoid arthritis. Genes Immun.

[34] Patterson BK, Seethamraju H, Dhody K, et al. Disruption of the CCL5/RANTES-CCR5 pathway restores immune homeostasis and reduces plasma viral load in critical COVID-19. Preprint. medRxiv. 2020;2020.05.02.20084673. 10.1101/2020.05.02.20084673. Published 2020 May 5.

[35] Kumar RN, Tanna SD, Shetty AA, Stosor V. COVID-19 in an HIV-positive kidney transplant recipient. Transpl Infect Dis. 2020:e13338. 10.1111/tid.13338. 10.1111/tid.13338 PMC7267082 32453483

[36] Panda AK, Padhi A, Prusty BAK (2020). CCR5 Δ32 minorallele is associated with susceptibility to SARS-CoV-2 infection and death: An epidemiological investigation. Clini Chim Acta.

[37] Qin C, Zhou L, Hu Z, et al. Dysregulation of immune response in patients with Coronavirus 2019 (COVID-19) in Wuhan, China. Clin Infect Dis. 2020;71(15):762–8. 10.1093/cid/ciaa248. 10.1093/cid/ciaa248 PMC7108125 32161940

[38] Zheng Z, Peng F, Xu B, et al. Risk factors of critical & mortal COVID-19 cases: a systematic literature review and meta-analysis. J Infect. 2020;81(2):e16–e25. 10.1016/j.jinf.2020.04.021. 10.1016/j.jinf.2020.04.021 PMC7177098 32335169

[39] Zheng M (2020). Functional exhaustion of antiviral lymphocytes in COVID-19 patients. Cell Mol Immunol.

[40] Kinne RW (2000). Macrophages in rheumatoid arthritis. Arthritis Res.

[41] Doorenspleet ME (2014). Rheumatoid arthritis synovial tissue harbours dominant B-cell and plasma-cell clones associated with autoreactivity. Ann Rheum Dis.

[42] Liao M (2020). Single-cell landscape of bronchoalveolar immune cells in patients with COVID-19. Nat Med.

[43] Wang C (2020). Alveolar macrophage dysfunction and cytokine storm in the pathogenesis of two severe COVID-19 patients. EBioMedicine.

[44] Wen W (2020). Immune cell profiling of COVID-19 patients in the recovery stage by single-cell sequencing. Cell Discov.

[45] Jafarzadeh A (2020). Contribution of monocytes and macrophages to the local tissue inflammation and cytokine storm in COVID-19: lessons from SARS and MERS, and potential therapeutic interventions. Life Sci.

[46] Liu L, Xu L, Lin C. T cell response in patients with COVID-19. Blood Sci. 2020;2(3):76–8. 10.1097/BS9.0000000000000050. 10.1097/BS9.0000000000000050 PMC8974945 35402822

[47] Meyerowitz EA (2020). Rethinking the role of hydroxychloroquine in the treatment of COVID-19. FASEB J.

